# Identification of novel cuproptosis-related lncRNA signatures to predict the prognosis and immune microenvironment of breast cancer patients

**DOI:** 10.3389/fonc.2022.988680

**Published:** 2022-09-20

**Authors:** Zi-Rong Jiang, Lin-Hui Yang, Liang-Zi Jin, Li-Mu Yi, Ping-Ping Bing, Jun Zhou, Jia-Sheng Yang

**Affiliations:** ^1^ Department of Surgical Oncology, Ningde Municipal Hospital of Ningde Normal University, Teaching Hospital of Fujian Medical University, Ningde, China; ^2^ Institute of Medical Biology, Chinese Academy of Medical Sciences and Peking Union Medical College, Kunming, China; ^3^ Department of Pathology, The First Affiliated Hospital of Guangdong University of Pharmacy, Guangzhou, China; ^4^ Academician Workstation, Changsha Medical University, Changsha, China; ^5^ School of Electrical & Information Engineering, Anhui University of Technology, Ma’anshan, China

**Keywords:** cuproptosis, breast cancer, lncRNA, tumor microenvironment, tumor mutation burden

## Abstract

**Background:**

Cuproptosis is a new modality of cell death regulation that is currently considered as a new cancer treatment strategy. Nevertheless, the prognostic predictive value of cuproptosis-related lncRNAs in breast cancer (BC) remains unknown. Using cuproptosis-related lncRNAs, this study aims to predict the immune microenvironment and prognosis of BC patients. and develop new therapeutic strategies that target the disease.

**Methods:**

The Cancer Genome Atlas (TCGA) database provided the RNA-seq data along with the corresponding clinical and prognostic information. Univariate and multivariate Cox regression analyses were performed to acquire lncRNAs associated with cuproptosis to establish predictive features. The Kaplan-Meier method was used to calculate the overall survival rate (OS) in the high-risk and low-risk groups. High risk and low risk gene sets were enriched to explore functional discrepancies among risk teams. The mutation data were analyzed using the “MAFTools” r-package. The ties of predictive characteristics and immune status had been explored by single sample gene set enrichment analysis (ssGSEA). Last, the correlation between predictive features and treatment condition in patients with BC was analyzed. Based on prognostic risk models, we assessed associations between risk subgroups and immune scores and immune checkpoints. In addition, drug responses in at-risk populations were predicted.

**Results:**

We identified a set of 11 Cuproptosis-Related lncRNAs (GORAB-AS1, AC 079922.2, AL 589765.4, AC 005696.4, Cytor, ZNF 197-AS1, AC 002398.1, AL 451085.3, YTH DF 3-AS1, AC 008771.1, LINC 02446), based on which to construct the risk model. In comparison to the high-risk group, the low-risk patients lived longer (p < 0.001). Moreover, cuproptosis-related lncRNA profiles can independently predict prognosis in BC patients. The AUC values for receiver operating characteristics (ROC) of 1-, 3-, and 5-year risk were 0.849, 0.779, and 0.794, respectively. Patients in the high-risk group had lower OS than those in the low-risk group when they were divided into groups based on various clinicopathological variables. The tumor burden mutations (TMB) correlation analysis showed that high TMB had a worse prognosis than low-TMB, and gene mutations were found to be different in high and low TMB groups, such as PIK3CA (36% versus 32%), SYNE1 (4% versus 6%). Gene enrichment analysis indicated that the differential genes were significantly concentrated in immune-related pathways. The predictive traits were significantly correlated with the immune status of BC patients, according to ssGSEA results. Finally, high-risk patients showed high sensitivity in anti-CD276 immunotherapy and conventional chemotherapeutic drugs such as imatinib, lapatinib, and pazopanib.

**Conclusion:**

We successfully constructed of a cuproptosis-related lncRNA signature, which can independently predict the prognosis of BC patients and can be used to estimate OS and clinical treatment outcomes in BRCA patients. It will serve as a foundation for further research into the mechanism of cuproptosis-related lncRNAs in breast cancer, as well as for the development of new markers and therapeutic targets for the disease.

## Introduction

Breast cancer (BC) is a cancer that affects mainly women and is a major factor in mortality worldwide (1). BC has overtaken lung cancer as the most usual cancers, with an estimated 2.26 million new cases in the word (11.7%) (2–4). Developing countries account for nearly 60% of mortality (4). Despite early screening and development of anticancer strategies, the prognosis of BC patients has improved significantly (5), but the recurrence rate of BC remains high (6, 7). The prognosis of breast cancer depends not only on the pathological stage at the time of detection but also mainly on the category of breast cancer (8). Breast cancer can be classified into different subgroups, which are primarily on the basis of human epidermal growth factor receptor 2 (HER2), progesterone receptor expression (PR), Ki-67 value, and estrogen receptor (ER). By exploring global gene expression profiles, breast cancer can be also divided into molecular subgroups, HER2-enriched, including Luminal B, Luminal A, Basal-like, and Normal-like (9). BC is a highly heterogeneous tumor, and the search for biomarkers which helps to the diagnosis, prognosis and prediction of breast cancer has important meaning for monitoring breast cancer recurrence and disclosing new therapeutic target spots throughout the treatment process (10, 11).

Cuproptosis is a new regulatory cell death pattern that mainly relies on the direct binding of fatty acyl components of the tricarboxylic acid (TCA) cycle of mitochondrial respiration, distinguished from other regulatory cell death features like pyroptosis, ferroptosis and apoptosis. With the accumulation of acylated protein and the subsequent decrease in iron-sulfur cluster protein, the cells died due to protein toxic stress. Copper is a vital cofactor for all organisms, but it can become poisonous if density exceed the threshold value maintained by evolutionarily conservative steady-state mechanisms. Current studies have found significant changes in copper content in serum and tumor tissues in some tumor patients (12–14). In addition, mechanisms regarding copper-dependent tumor growth and progression have been recently discovered and summarized in other studies (15, 16). Copper is also capable of promoting angiogenesis, which is essential for tumor metastasis and progression. Overload of copper can also lead to cell death. Because copper plays a crucial part in the occurrence, severity and development of cancer, it may be an important hub for halting cancer development (17).

Long non-coding RNA (lncRNA) means a non-coding RNA that is more than 200 nucleotides in length (18, 19). Increasing evidence show that LncRNA play a crucial role in regulating tumor occurrence and metastasis (20). For example, it has been demonstrated that the M2 polarization of macrophages and the aggressiveness of BC cells are both influenced by the LINC01140/miR-140-5p/FGF9 axis (21). LncRNA PVT1 can accelerate malignant changes in the phenotype of BC cells by controlling the MIRI-194-5P/BCLAF1 axis as a competing endogenous RNA (22). lncRNA were the key factors for BC chemical resistance (23). Although there are more and more studies on the role of lncRNA in cancer, our understanding of the role of lncRNA in the occurrence is less. Currently, there is a small number of studies on the lncRNA related to cuproptosis, and no studies on the lncRNA related to cuproptosis in BC.

In this study, we established a predictive features and internal validation on the basis of cuproptosis-related lncRNAs. We further analyzed its underlying value in predicting the diagnosis, prognosis, chemotherapy response, and tumor immune infiltration of patients with BC.

## Materials and methods

### Patients and datasets

We do this from the TCGA official website (https://portal.gdc.cancer.gov/); Data were obtained for 1089 alive patients with lncRNA expression. Fifty-two genes related to cuproptosis were downloaded from the genecard database and previous literature (24) (GeneCard filtering condition: Relevance score >20).

### Construction of prediction features of cuproptosis-associated lncRNA

Associations between cuproptosis-related genes and lncRNAs were calculated using the “limma” package in R. Using correlation coefficients |R2|> 0.35 and p < 0.05 as screening standard, in sum, 1,136 cuproptosis-related lncRNA expressions were obtained. We made use of univariate Cox regression analysis to gain cuproptosis-related lncRNA associated with the prognosis of BC patients, then constructed training and test cohorts according to the 1:1 random assignment principle. The training cohort was subjected to lasso-Cox regression analysis to acquire cuproptosis-related lncRNA, and the prediction features of cuproptosis-related lncRNA were constructed. The equation used for this analysis was as follows: Risk score = (Expi × βi). (Exp: expression level of model gene; β: model gene coefficient).

### Construction of nomogram

By combining the risk score with the clinicopathological characteristics of patients’ age, phase, and TNM stage, a nomogram that can forecast patients with BRCA’s 1-, 3-, and 5-year survival was developed. We verified that predicted survival is consistent with actual survival by using a calibration curve.

### Collection and pretreatment of epigenetic mutation data

Somatic changes in the BRCA cohort were obtained from the TCGA database. TMB was defined as the number of somatic, coding, base substitution, and indel mutations per megabase in the genome tested using non-synonymous and transcoding indels at the 5% limit of detection. The “MAF Tools” R package (25) has been used to test the amount of somatic non-sense point mutations in per sample. Reveals how BRCA drives somatic changes in genes for samples with low and high-risk scores.

### Gene ontology and Kyoto Encyclopedia of genes and genome pathway analysis

In accordance with the median risk score, patients with BC were distinguish between high-risk team and low-risk team. The error detection rate (FDR) < 0.05 and | log2fold change (FC) > 1| were used as screening standard for obtaining cuproptosis-related genes (DEGs) with divergence in expression. We utilize the “ggplot2” package for GO and KEGG analysis. The difference was regarded as statistically significant when the P value was less than 0.05.

### Correlation between risk score and immune cell infiltration

The relative ratio of infiltrating immune cells was calculated using the ssGSEA and CIBERSORT R scripts. In exploring the correlation during risk rating values and immunologically infiltrating cells, we used a Spearman rank correlation analysis.

### Role of predictive features in predicting clinical treatment outcome

To assess the role of predictive features in forecasting response to BRCA therapy, we computed the maximum inhibitory concentration (IC50) half of the common chemotherapeutic agents used to the clinical treatment of BC. In order to compare the IC50 values between high-risk and low-risk teams, the Wilcoxon signed rank test was used.

### Data analysis

All data analyses were executed using the R software (version 4.0.2). The expression of cuproptosis-related DEGs in both normal and cancer tissues was examined using Wilcoxon’s test. Using univariate Cox regression analysis, the association between cuproptosis-related lncRNA and overall survival (OS) was examined, and lasso- Cox analysis was applied to sift cuproptosis-related lncRNA to set up predictive features. The Kaplan-Meier approach and the logarithmic rank examine were used to compare the OS of patients in the high-risk team and the low-risk team. The ROC curve was plotted using the “survivalROC” package, and the area under the curve (AUC) value was calculated.

## Results

### Construction of prediction features of cuproptosis-associated lncRNA

**Figure 1:**
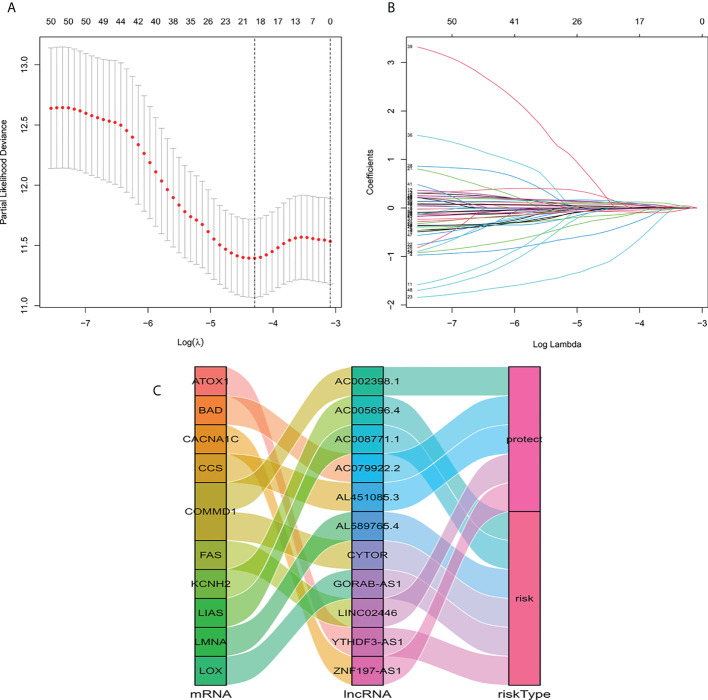
Construction of a signature for predicting features of cuproptosis-related lncRNA using the Lasso method. **(A)** Cross validation diagram. **(B)** LASSO coefficients of prognostic genes. **(C)** Sankey diagram of prognostic cuproptosis-related lncRNAs.

We identified 1136 lncRNA associated with cuproptosis (**Supplementary Table S1**). Univariate Cox regression analysis showed there were 51 lncRNA related to the recovery results of BC patients. We made use of Lasso multivariate Cox regression analysis to select for efficient prognostic-related symbols, and a prognostic model was established from the training set (**Figures 1A, B**). It showed that 11 lncRNA (GORA B-AS1, AC 079922.2, AL 589765.4, AC 005696.4, Cytor, ZNF 197-AS1, AC 002398.1, AL 451085.3, YTHDF 3-AS1, AC 008771.1, LINC 02446) associated with cuproptosis were identified to construct predictive signatures. GORAB-AS1, AL589765.4, AC005696.4, CYTOR, YTHDF3-AS1, AC008771.1 were protective while AC079922.2, ZNF197-AS1, AC002398.1, AL451085.3, LINC02446 were risk factors (**Figure 1C**). The risk grade was calculated as follows: Risk grade = (0.643×GORAB-AS1 expression) +(-0.892×AC079922.2 expression) +(0.160×AL589765.4 expression) +(0.160×AC005696.4 expression) +(0.126×CYTOR expression) +(-1.927×ZNF197-AS1 expression) +(-0.278×A C002398.1 expression) +(-0.603×AL451085.3 expression) +(0.206×YTHDF3-AS1 expression) +(0.055×AC008771.1 expression) +(-0.147×LINC02446 expression).

### Relationship between predictive features and prognosis of BC patients

**Figure 2:**
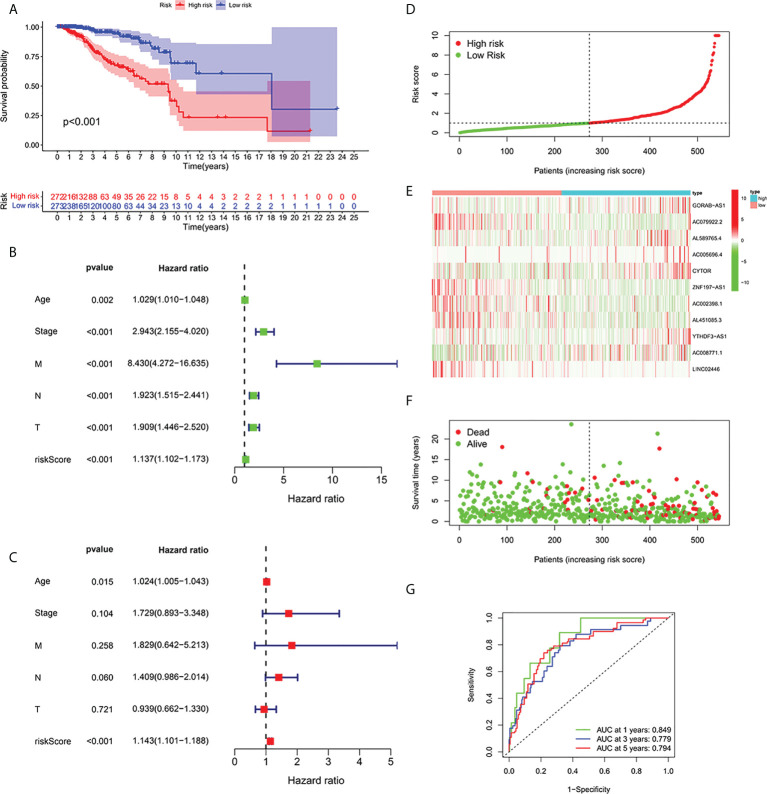
Relationship between prognosis and predictive features of patients with BC. **(A)** OS rates for BC patients in the high-risk and low-risk teams according to a Kaplan-Meier analysis. **(B)** Forest map of univariate Cox regression analysis. **(C)** Forest map of multivariate Cox regression analysis. **(D)** Risk score of BRCA patients calculated according to the model and division of high- and low-risk teams. **(E)** Gene expression heat maps. **(F)** Survival status. **(G)** ROC curve of the predictive characteristic and AUC of 1-, 3-, and 5-year survival. OS, overall survival; ROC, receiver operating characteristics; AUC, area under curve; T, tumor; N, lymph nodes; M, Metastasis.

According to the formula, each patient’s risk grade was determined, and the patients were then divided into high-risk and low-risk teams based on the median risk score. To ascertain the value of the risk score in predicting the prognosis of BC patients, the OS time in the high-risk and low-risk groups was analyzed using the Kaplan-Meier method. The OS time in the high-risk team was significantly reduced when compared to the low-risk team (**Figure 2A**, p<0.001). To ascertain if predictive features were an independent prognostic element in patients with BC, Cox regression analysis was executed. Age, T stage, N stage, M stage, and risk score were found to be significantly correlated with OS in univariate Cox regression analysis of BC patients (**Figure 2B**). In patients with BC, multivariate Cox regression analysis revealed that risk scores and age were independent predictors of OS (**Figure 2C**). Risk scores for high and low risk teams as displayed in **Figures 2D–F**. As shown in the figure, the number of patients who died increased as the risk increased, and the expression of Cuproptosis-related lncRNA in the two risk groups was also shown. Indicating good predictive performance, the 1-, 3-, and 5-year survival AUCs were 0.849, 0.779, and 0.794, respectively (**Figure 2G**).

### Identification and verification of nomograms

**Figure 3:**
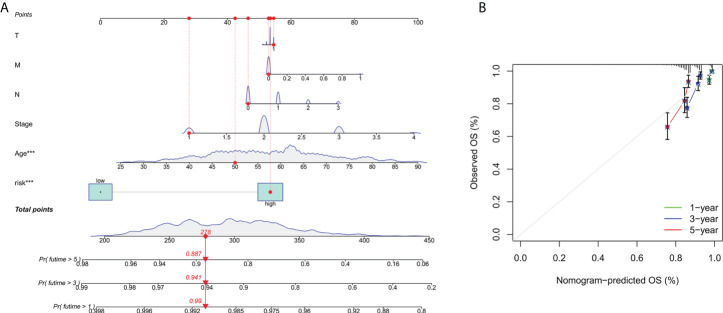
Construction and verification of nomograms. **(A)** The 1,3and 5 years OS of patients with BC is predicted by nomograms that combine clinical pathology variables with risk scores. **(B)** The calibration curve tested the agreement between the actual OS rate and the predicted 1-,3-, and 5-year survival rates.

To further forecast the prognosis of patients with BC, we set up an nomogram with clinical pathology variables and risk scores that predicts the 1, 3, and 5 years prognosis of patients with BC (**Figure 3A**). The calibration curve demonstrates good agreement between the predicted survival at 1, 3, and 5 years and the actual OS rates (**Figure 3B**). All results suggest that histograms created by cuproptosis-related lncRNAs have good prognostic potential for BC patients.

### The correlation between predictive features and prognosis of BC patients with different clinical pathological variables

**Figure 4:**
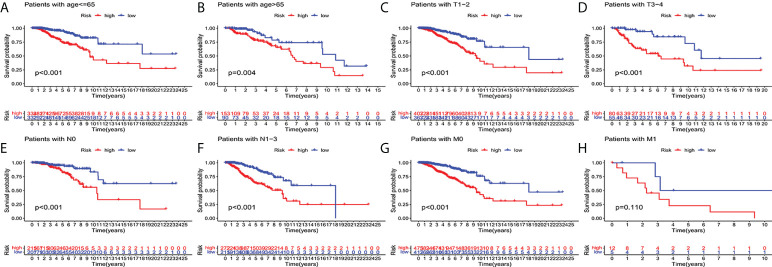
Kaplan-Meier survival curves of high-risk and low-risk teams in the patients sorted by different clinical pathological variables. **(A, B)** age. **(C, D)** T staging. **(E, F)** N staging. **(G, H)** M staging. T, tumor; N, lymph node; M, distant transfer.

In order to investigate the correlation between prognosis and predictive features of patients with BC classified in accordance with different clinical pathological variables, patients with BC were grouped according to age, T stage, N stage and M stage. Significantly fewer patients in the high-risk group survived longer than those in the low-risk group. These findings imply that regardless of clinicopathological factors, the predictive function can forecast the prognosis of BC patients (**Figures 4A–H**).

### Validation of predictive signature

**Figure 5:**
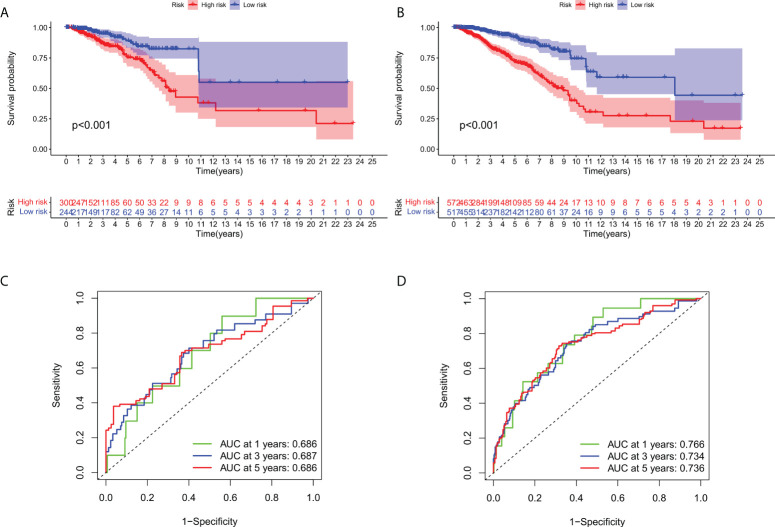
Internal verification of OS prediction signatures based on the TCGA dataset. **(A)** Kaplan-Meier survival curves in the test cohort. **(B)** Kaplan-Meier survival curves in the total cohort. **(C)** ROC curves and AUC for 1-, 3-, and 5-year survival in the test cohort. **(D)** ROC curves and AUC for 1-, 3-, and 5-year survival in the total cohort.

To verify of the applicability of OS prediction features on the basis of the entire TCGA dataset, validation in both the test cohort and the total cohort, the test cohort showed that patients in the high-risk group had lower OS ratios than those in the low-risk group (**Figure 5A**, p < 0.001). The high-risk team’s prognosis was worse than the low-risk team’s prognosis for the entire cohort (**Figure 5B**, p < 0.001). The ROC curves of both cohorts showed cracking predictive performance. In the test cohort, the AUC was 0.686, 0.687, and 0.686 for the 1-, 3-, and 5-year survival rates, separately (**Figure 5C**). In the total cohort, the 1-, 3-, and 5-year survival AUCs were 0.766, 0.734, and 0.736, separately (**Figure 5D**).

### Association between risk signature and TMB

**Figure 6:**
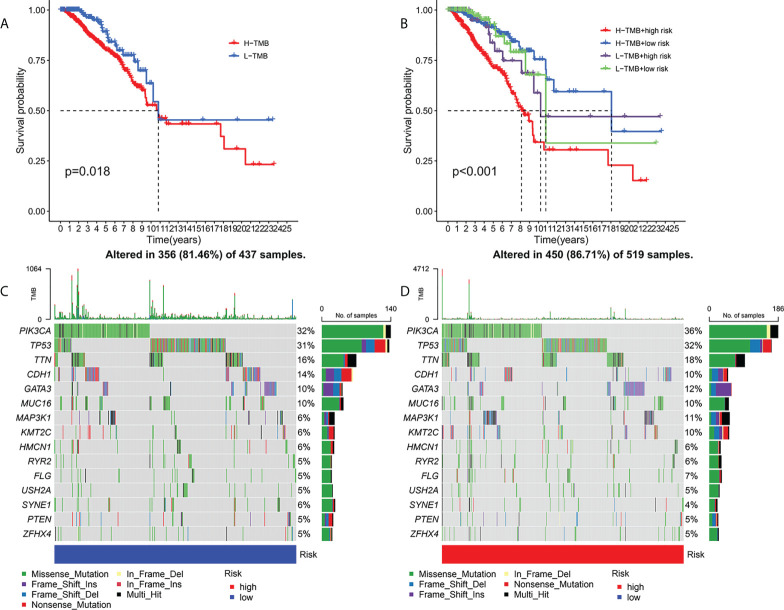
Association of risk score with TMB and gene mutations. Kaplan-Meier curves for high and low TMB teams **(A)**. Kaplan-Meier curve for patients layered by TMB and risk score **(B)**. OncoPrint was built with a low-risk score **(C)** and a high-risk score **(D)**.

Current studies had emphasized that high TMB were significantly related with abundant CD8+ T cells, which can recognize cancer cells and then lead to an anti-tumor immune outcome (26–29). Therefore, we inferred that TMB might serve as a non-negligible prognostic element in the anti-tumor immunotherapy response, aiming to study interactions between risk scoring and TMB to reveal the genetic variation of subtypes of risk scoring (30). Patients were distributed to different subtypes on the TMB immune set point line. The survival curve indicated that a high TMB value markedly indicated a short total survival time (p = 0.018, **Figure 6A**). To further quest for validity of risk scores and consistent prognostic significance of TMB, we tested and verified the synergistic influence of the two markers in predicting the prognosis of BRCA. As shown by the layered survival curve, the TMB status did not interfere with the prognostic performance of the risk score. The risk score subgroup showed significant difference in prognosis between the low and high TMB status hypotypes (p < 0.001, **Figure 6B**).

In addition, we probed and visualized the distribution of gene mutations among subtypes with different risk scores. The integrated scenery of somatic variations showed mutational models and clinical features of the top 15 most frequently changed driving genes (**Figures 6C, D**). Significant mutation (SMG) profiles indicated that PIK3CA (36% versus 32%) had a higher rate of somatic mutations in the high-risk core subtypes, while SYNE1(4% versus 6%) had a higher rate of somatic mutations in the low-risk core subtypes—a subset of risk scores. These findings may shed new light on the intrinsic link between high and low risks and somatic variation in BRCA immunotherapy. All in all, these consequences suggest that risk scores may serve as independent prognostic predictors and have the potential to access clinical outcomes of anti-tumor immunotherapy.

### Heat map and gene enrichment analysis

**Figure 7:**
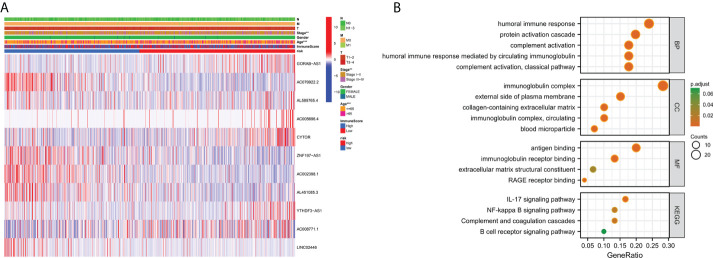
Clinically relevant heat map and GO/KEGG pathway enrichment analysis. **(A)** On the basis of risk characteristics associated with prognosis, a heat map of distribution of cuproptosis-related lncRNA and clinical pathology variables was plotted. The more intense the red, the more intense the expression. The more intense the blue, the more subdued the expression. **p < 0.01, ***p < 0.001. **(B)** The graph depicts the GO and KEGG analysis of differential genes with high and low risk..

To investigate the expression of prognostic model genes in clinical characteristics, we established expression heatmaps based on clinical features, correlations between high-risk and low-risk groups, patient age, tumor stage, and lymphoid. Prognostic model genes between nodes were investigated. Lymph node metastasis and Immune Score (**Figure 7A**). Since the prognosis of patients in the high-risk team and the low-risk team was different, enrichment analysis was executed to study possible discrepancies between the high-risk team and the low-risk team (**Figure 7B**). Moreover, we discovered that in biological process (BP), enriched in humoral immune response, protein activation cascade, completion activation, classical pathway. Human immune response mediated by circulating immune globulin, the cellular component is enriched in the immune globulin complex, circulating, blood microparticle, external side of plasma membrane, collagen-containing extracellular matrix. Molecular-rich antigen binding, rage receptor binding, immunoglobulin receptor binding, extra-granular matrix structural constitution. More importantly, KEGG was enriched in IL-17 signaling pathway-κB signaling pathway, B cell receptor signaling pathway, completion and colonization cascades.

### Immune cell infiltration and immune-related function

**Figure 8:**
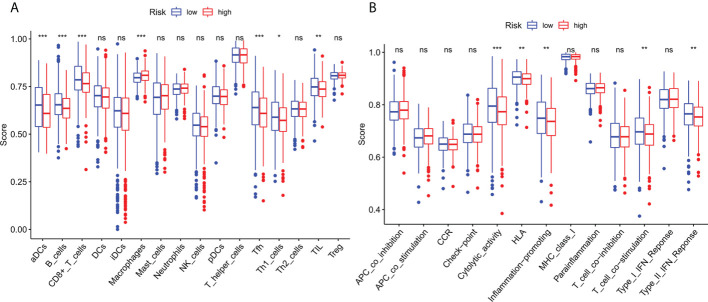
Immune infiltration cell score and immune-related function in high-risk and low-risk population. **(A)** The ssGSEA algorithm was used to calculate the levels of infiltration of 16 immune cells in high-risk and low-risk populations. **(B)** Relationship between predictive features and 13 immune-related functions. *p< 0.05; **p< 0.01; ***p< 0.001; ns, not significant.

To investigate further the relationship between risk scores and immune cells and function, we used ssGSEA to calculate enrichment scores for various immune cell subsets, associated functions, or pathways. Results showed that sensitized Dendritic cells (aDC), B cells, CD8+T cells, T follicle helper cells (Tfh) cells, T helper type 1 (Th1) cells, Tumor Infiltrating Lymphocytes (TIL) were strikingly different between the high-and low-risk teams (**Figure 8A**). The IFN response was lower in the high-risk team than in the low-risk team for cytolytic activity, Human Leukocyte Antigen (HLA), T-cell-co-stimulation, inflammation promotion, and Type II immune function scoring (**Figure 8B**). These findings suggest that immune function is more inactive in high-risk groups.

### Correlation between predictive characteristics and BC treatment

**Figure 9:**
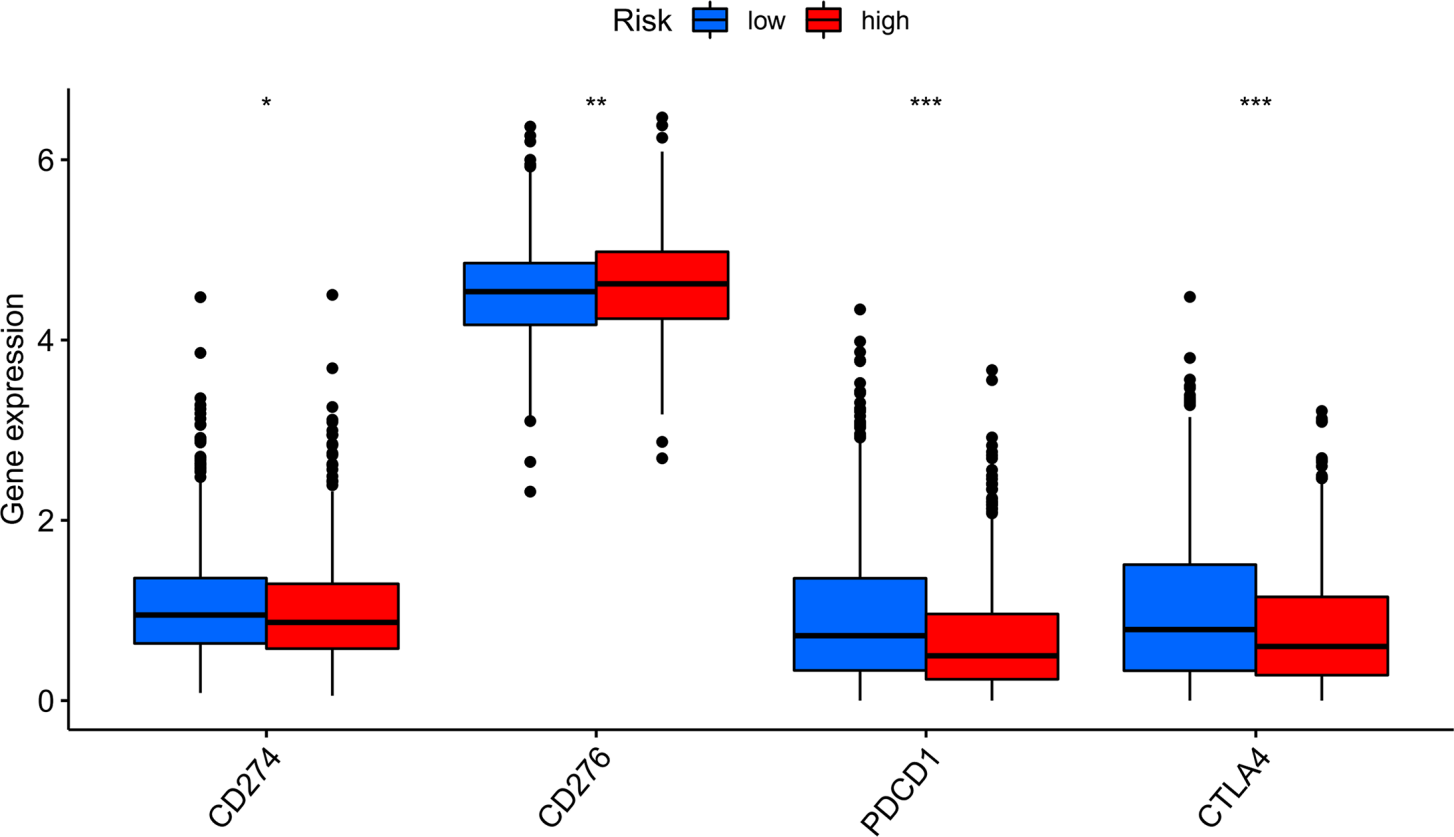
Immune checkpoint expression in BRCA patients from two different risk groups. Expression of two immune checkpoints (CD274, CD276, PDCD1 and CTLA4) in the TCGA cohort. ANOVA was applied as significance test, * P <0.05, ** P <0.01, *** P <0.001.

**Figure 10:**
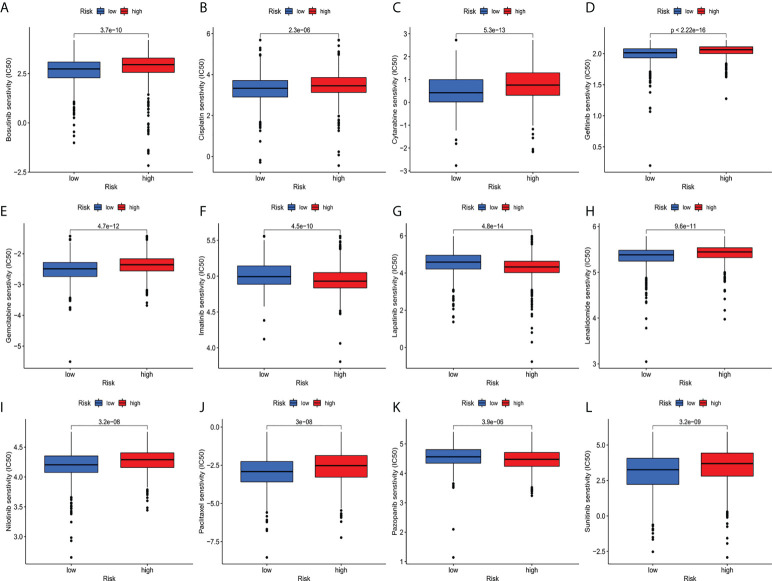
**(A–L)** IC50 for small molecule drugs in high and low risk populations.**(A)**: Bosutinib, **(B)**: Cisplatin, **(C)**: Cytarabine, **(D)**: Gefitinib, **(E)**: Gemcitabine, **(F)**: Imatinib, **(G)**: Lapatinib, **(H)**: Lenalidomide, **(I)**: Nilotinib, **(J)**: Paclitaxel, **(K)**: Pazopanib, **(L)**: Sunitinib. IC50, half maximum inhibitory concentration.

Compared with the low-risk team, CD276 expression was significantly increased in the high-risk group, suggesting that high-risk patients may respond to anti-CD276 immunotherapy. The expression levels of CD274, PDCD1, and CTLA4 in the low-risk team were significantly increased, suggesting that low-risk patients might have a potential response to immunotherapy with PD-1, PD-L1, and CTLA4 (**Figure 9**). In addition to immunotherapy, we also studied the relationship between predictive features and the general chemotherapeutic response of BC. The IC50 values of imatinib, lapatinib, and pazopanib in the high-risk team were found to be lower, while those of bosutinib, cisplatin, cytarabine, gefitinib, gemcitabine, lenalidomide, nilotinib, paclitaxel, and sunitinib in the high-risk team were found to be higher (**Figures 10A–L**), which helped explore treatment options for high-risk and low-risk populations.

## Discussion

Although the mortality rate of BC has decreased due to the early detection and advanced treatment, the morbidity rate continues to increase annually (31–33). The role of cuproptosis in cancer was complex. Significant changes in copper content in serum and tumor tissues of patients with different cancers (such as breast cancer, cervical cancer, thyroid cancer, lung cancer, pancreatic cancer, prostate cancer, ovarian cancer, oral cancer, breast cancer, and bladder cancer) had been found (12–14). More and more studies had discovered that cuproptosis played a crucial role in the occurrence and development of cancer. However, resent research mainly paid attention to the role of cuproptosis in cancer mechanisms (12–14) and few studies to its role in cancer prognosis.

Researches had shown that lncRNA do not represent transcriptional noise. which played an essential role in tumors (34–37). For example, autophagy-related lncRNA characteristics could accurately forecast the prognosis of patients with Bladder Cancer (38). Cuproptosis-associated lncRNA was a good predictor of prognosis in patients with colon cancer (39). Prognosis of patients with BC had not been studied by constructing predictive features of lncRNA associated with cuproptosis (40, 41). Therefore, it was important to identify predictive features of lncRNA associated with cuproptosis in BC patients. In this research, we applied univariate Cox regression analysis to analyze the relationship between cuproptosis-related lncRNA and the prognosis of patients with BC patients and discovered that 51 lncRNA were related to the prognosis of patients with BC. Through lasso multivariate Cox regression analysis, we determined that 11 lncRNAs associated with cuproptosis were included in the predictive signature.

We also found significant co-expression of mRNA (LOX, BAD, LMNA, KCNH2, COMMD1, CACNA1C, COMMD1, CCS, ATOX1, LIAS, FAS) with these lncRNA. Among them, LIAS was a vital driving factor for the death of copper (24). At present, some clinical data indicate the correlation between genetic changes and immunotherapy response (42, 43). We computed and identified TMB, which was a predictor of immunotherapeutic sensitivity and increases significantly with increased risk scores. Succedent stratified survival curves indicated that the risk scores had prognostic capabilities independent of TMB, indicating that TMB and risk scores represented various aspects of immunobiology. Furthermore, the risk score, together with the mutation data, revealed a prominent discrepancy in the frequency of gene variation at the transcriptome level between different groupings. In this work, the mutation rate of PIK3CA was significantly increased in subtypes with high-risk scores, while the mutation rate of SYNE1 was increased in patients with low-risk scores.

Since the prognosis of patients in the high-risk team and the low-risk team was different, enrichment analysis was enforced to study possible divergences between the high-risk team and the low-risk team. Besides, we discovered that in biological process (BP), enriched in humoral immune response, protein activation cascade. The cellular component was enriched in the immune globulin complex and immunoglobulin receptor binding. More importantly, KEGG was enriched in IL-17 signaling pathway, completion and colonization cascades, NF-κB signaling pathway. Enrichment results showed that differential genes with high risk were closely linked to tumor and immune-related pathways.

Subsequently, ssGSEA results indicated that activated dendritic cells (ADCs), CD8+T cells, B cells, and tumor-infiltrating lymphocytes (TILs) were markedly different between the high-risk and low-risk teams. The IFN response was lower in the high-risk team than in the low-risk team for cytolytic activity, human leukocyte antigen (HLA), and Type II immune function score, inflammation promotion. These results of the survey suggested that immune function is more inactive in high-risk teams. Aside from increased tumor immune cell infiltration, the high-risk group was associated with decreased antitumor immunity, and HLA and type I IFN responses scored lower in the high-risk teams. Therefore, in breast cancer, reduced antitumor immunity in high-risk teams could explain the poor prognosis. Our study also indicated that high-risk patients may be susceptible to anti-CD276 immunotherapy and conventional chemotherapy drugs imatinib, lapatinib, and pazopanib, but resistant to bosutinib, cisplatin, cytarabine, gefitinib, gemcitabine, lenalidomide, nilotinib, paclitaxel, and sunitinib. This proved that high-risk individuals can obtain a benefit from the combined immunotherapy and chemotherapy, providing a basis for precise and individualized treatment of BC patients.

However, there were some limitations in our study. Firstly, we only used data from the TCGA database for internal validation, and we needed data from other databases for external validation to test the predictive signature’s applicability further. Secondly, the mechanism of action of cuproptosis-related lncRNA in BC needed to be further verified through experiments.

## Conclusion

The cuproptosis-related lncRNA signature, in conclusion, can independently predict a patient’s prognosis for BC and provides evidence for a potential cuproptosis-related lncRNA mechanism in BC and its response to clinical therapy.

## Data availability statement

The original contributions presented in the study are included in the article/**Supplementary Material**. Further inquiries can be directed to the corresponding authors.

## Ethics statement

Ethical review and approval was not required for the study on human participants in accordance with the local legislation and institutional requirements. Written informed consent for participation was not required for this study in accordance with the national legislation and the institutional requirements.

## Author contributions

J-SY and JZ conceived and designed the study. Z-RJ, P-PB, and L-HY performed the experiments. L-ZJ and L-MY analyzed the data. Z-RJ wrote the manuscript. All authors contributed to the article and approved the submitted version.

## Acknowledgments

The authors thank reviewers for helpful comments on the manuscript.

## Conflict of interest

The authors declare that the research was conducted in the absence of any commercial or financial relationships that could be construed as a potential conflict of interest.

## Publisher’s note

All claims expressed in this article are solely those of the authors and do not necessarily represent those of their affiliated organizations, or those of the publisher, the editors and the reviewers. Any product that may be evaluated in this article, or claim that may be made by its manufacturer, is not guaranteed or endorsed by the publisher.
